# Publishing in Ageing & Society: Top Ten Tips From the Editor-in-Chief

**DOI:** 10.1093/geroni/igaf122.1466

**Published:** 2025-12-31

**Authors:** Philip Taylor, Sandra Torres

**Affiliations:** University of Warwick, Coventry, England, United Kingdom; Uppsala University, Uppsala, Uppsala Lan, Sweden

## Abstract

Understanding the differences between academic writing genres is crucial to nailing down the craft that is writing for peer-review journal publication. Thus, this presentation will bring attention to the different publication outlets there are, the different readers these cater to, and how these differences impact how we craft manuscripts about the research we conduct. Using this as a starting point, as well as allusions to how data collected in specific national settings can be presented so that it can be used to contribute to ongoing international debates in social gerontology, this presentation will offer insight into an editor-in-chief’s top 10 tips. By bringing specific attention to the routines that established international peer-review journals, such as Ageing & Society utilize, and the expectations that editors place on the different sections of a manuscript, this presentation aims to give attendees valuable insights into how to write for publication in such journals.

